# Educational outcomes associated with persistent speech disorder

**DOI:** 10.1111/1460-6984.12599

**Published:** 2021-02-02

**Authors:** Yvonne Wren, Emma Pagnamenta, Tim J. Peters, Alan Emond, Kate Northstone, Laura L. Miller, Sue Roulstone

**Affiliations:** ^1^ Bristol Speech and Language Therapy Research Unit, North Bristol NHS Trust University of Bristol Bristol UK; ^2^ Bristol Dental School University of Bristol Bristol UK; ^3^ School of Psychology and Clinical Language Sciences University of Reading Earley Gate Reading UK; ^4^ Population Health Sciences, Bristol Medical School Bristol UK; ^5^ Faculty of Health and Applied Sciences University of the West of England Bristol UK

**Keywords:** speech disorder, education, outcomes, children, ALSPAC

## Abstract

**Background:**

Children with persistent speech disorder (PSD) are at higher risk of difficulties with literacy, with some evidence suggesting an association with poorer educational attainment. However, studies to date have either used small clinical samples, which exclude children who have not been referred to clinical services, or relied on parent–teacher report of children's speech development. There is a need for an inclusive study to investigate the impact of PSD on educational outcomes using a population‐based sample and robust measures of speech development.

**Aim:**

Using a large prospective UK population‐based study—the Avon Longitudinal Study of Parents and Children (ALSPAC)—this study investigated: (1) how children identified with PSD at age 8 years perform on educational attainment tests at ages 10–11 and 13–14 years in comparison with children without PSD; and (2) whether children identified with PSD at age 8 years are more likely to receive a label of special educational needs (SEN) in secondary school.

**Methods & Procedures:**

We examined the data for 263 children with PSD and 6399 controls who had speech assessed at age 8 years in a research clinic. Educational attainment was measured using data from English school standard attainment tests. Data on SEN categorization were obtained between 11 and 13 years of age. Children with PSD and controls were compared using regression analyses adjusted for biological sex, maternal age, verbal, performance and full‐scale IQ.

**Outcomes & Results:**

Children with PSD at age 8 years were more likely to achieve lower attainment scores at ages 10–11 years in English and mathematics and across all three subjects of English, mathematics and science at ages 13–14 years after controlling for biological sex and maternal education; score below target levels for English at both time points after controlling for verbal IQ, and at ages 13–14 years after controlling for performance IQ; and receive a label of SEN (typically for the category of cognition and learning needs or communication and interaction needs) in secondary school.

**Conclusions & Implications:**

PSD identified at age 8 years is associated with poor educational attainment at ages 10–11 and 13–14 years in the core subjects of English, mathematics and science. Children with PSD at age 8 years are more likely to be identified with SEN at ages 11–13 years, particularly cognition and learning needs, and communication and interaction needs. We need to be aware of the potential for the long‐term impact of PSD on educational attainment in providing appropriate and effective support throughout school.

**What this paper adds**

**What is already known on the subject**

- Speech‐sound disorder is associated with reading and spelling difficulties, with some evidence to suggest that PSD is associated with a higher risk of literacy difficulties. Limited evidence also suggests that speech‐sound disorder may be associated with poorer educational attainment. However, studies to date have used small clinical samples or parent–teacher report of speech development and there is a need to determine whether the association is observed in larger and more inclusive population‐based samples.

**What this paper adds to existing knowledge**

- This prospective, longitudinal study of a large community‐based sample of English children has shown that PSD is associated with poorer educational attainment at the end of primary school and at ages 13–14 years. Children with PSD are also more likely to be identified as having SEN in secondary school, especially communication and interaction needs but also including cognition and learning needs.

**What are the potential or actual clinical implications of this work?**

- Understanding the long‐term implications of PSD on educational attainment highlights the importance of ongoing monitoring and support to enable children to reach their potential throughout primary and secondary school. The identification of children with a history of PSD during transition to secondary school will enable effective support to be put in place. The intervention for children with PSD should involve close collaboration between speech and language therapists and education professionals.

## Introduction

Difficulties producing speech sounds in early childhood are common (Broomfield and Dodd 2004a), with reported rates of speech‐sound disorder (SSD) ranging from 2.3% to 24.6% (Beitchman *et al*. 1986, Eadie *et al*. 2015, Jessup *et al*. 2008, Law *et al*. 2000, Shriberg *et al*. 1999) depending on the age, definition and measure used. SSD is defined as ‘any combination of difficulties with perception, articulation/motor production, and/or phonological representation of speech segments (consonants and vowels), phonotactics (syllable and word shapes), and prosody (lexical and grammatical tones, rhythm, stress, and intonation) that may impact speech intelligibility and acceptability’ (International Expert Panel on Multilingual Children's Speech 2012), and can include difficulties with phonological knowledge of speech sounds and difficulties with the motor‐based production or articulation of sounds (Eadie *et al*. 2015).

Most native speakers of English are able to use the full range of vowels and consonants by 8 years of age (Dodd *et al*. 2003), and SSD prevalence rates decrease in older childhood (Keating *et al*. 2001, McKinnon *et al*. 2007), suggesting that for many this is a transient condition. However, findings from a prospective population‐based study found that 3.6% of 8‐year‐olds have SSD, indicating that this is a persistent problem for some children whose difficulties remain after the period of typical speech acquisition (Wren *et al*. 2016). This is backed up by clinical research, which showed that 8.8% of speech and language therapy referrals for speech difficulties in an area in the north‐east of England were for children over the age of 7 years (Broomfield and Dodd 2004b).

Persistent speech disorder (PSD) can be defined as SSD in children who are beyond the age of typical speech acquisition. In that sense, it can be viewed as a subtype of SSD that is determined by age rather than error types. Definitions vary, however, with some reports in the literature including children with isolated speech errors (sometimes referred to as common clinical distortions (Shriberg 1993), such as lateralized or dentalized production of /s/or distortions of /ɹ/), and others excluding them on the basis that this group follows a different trajectory to those who demonstrate more variation in their speech production (Wren *et al*. 2012). Typically, children with PSD show little spontaneous improvement and a poor response to intervention and are more likely to have a combination of phonological and articulatory errors (Wren *et al*. 2012). Moreover, prospective longitudinal studies have identified several predictors of PSD, including male biological sex, family history, atypical errors, lower socioeconomic status, hearing loss and suspected coordination difficulties (Eadie *et al*. 2015, Morgan *et al*. 2017, Wren *et al*. 2016).

Whilst these findings are important in helping us to understand the nature of PSD and what co‐morbidities may occur, it is vital that we understand what impact PSD has on children growing up and whether there are long‐term consequences which need to be identified and addressed. In particular, there is a need to identify how problems with speech development may affect children's educational outcomes such as progress in school and educational attainment, given the impact that these can have on well‐being, employment and life chances. Knowledge of educational outcomes that are potentially associated with PSD can help inform and ensure that children with PSD receive the support and intervention they need to reach their potential, for example, identifying and monitoring through transitions and raising awareness of difficulties children with PSD might face in terms of literacy and wider educational development.

### PSD and literacy development

Previous research addressing educational outcomes of speech difficulties in children has largely focused on a broader group of children with SSD, rather than PSD specifically. Thus, there is evidence to show that children with SSD are more likely to have difficulties with the acquisition of literacy skills than typically developing children (Anthony *et al*. 2011, Holm *et al*. 2008, McCormack *et al*. 2009). Longitudinal studies have found that children with SSD are at higher risk of reading and spelling difficulties at primary school age (Lewis *et al*. 2000, Peterson *et al*. 2009). Moreover, studies carried out across the lifespan have suggested that SSD in childhood may be associated with difficulties with literacy in adulthood, many years after diagnosis (Felsenfeld *et al*. 1994, Lewis *et al*. 2007, Preston and Edwards 2007).

Difficulties with reading and spelling are thought to be linked to poor phonological awareness skills (Bird *et al*. 1995, Nathan *et al*. 2004a, Overby *et al*. 2012, Rvachew 2007, Tambyraja *et al*. 2020); for example, children with moderate to severe SSD are more likely to have difficulties accessing and/or storing phonological representations than typically developing children (Sutherland and Gillon 2007). Some studies have suggested that atypical speech sound errors may increase the risk of phonological awareness difficulties and poorer reading and spelling outcomes (Dodd *et al*. 1995, Holm *et al*. 2008, Leitao *et al*. 2000, Leitao and Fletcher 2004, Preston and Edwards 2010). However, the evidence is contradictory, as not all children with SSD have shown difficulties with phonological awareness or later literacy skills (Hesketh 2004, Leitao *et al*. 1997, Leitao and Fletcher 2004, Nathan *et al*. 2004a, Rvachew 2007).

There is however some evidence that those children who have persistent difficulties with their speech are at greater risk of reading and spelling difficulties (Nathan *et al*. 2004a, Preston and Edwards 2007, Tambyraja *et al*. 2020). A systematic review of studies published between 1998 and 2008 found that SSD was associated with difficulties with learning to read, reading, learning to write and writing, with a greater risk of such difficulties if the speech difficulties persisted into school age (McCormack *et al*. 2009). The ‘critical age hypothesis’ suggests that this is because speech difficulties are present as the child is learning to read (Bishop and Adams 1990, Nathan *et al*. 2004a). In a study of 92 children across four different schools in Los Angeles, children who performed more poorly on assessments of early reading skills (measures of phonological awareness) were more likely to make more speech errors at age 5 years, and make more atypical speech errors than children who were not delayed in reading skills, suggesting that concurrent difficulties with speech whilst a child is learning to read may have an impact on literacy development. Improvement in speech production was associated with reading skills at the end of the school year (Foy and Mann 2012). Children in this study were part of a larger intervention study targeting children at risk of reading difficulties and were not therefore representative of the general population.

In some studies, samples have included children who have SSD and developmental language disorder. These have shown that problems with literacy development are apparent for children with SSD, whether they have additional language problems or not (Bird *et al*. 1995) or that children with co‐occurring SSD and language problems show worse performance than those with SSD alone (Bishop and Clarkson 2003, Nathan *et al*. 2004a). However, sample sizes for these analyses have typically been small (Bird *et al*. 1995, Bishop and Clarkson 2003, Nathan *et al*. 2004a) or limited to SSD identification in younger age groups (Lewis *et al*. 2000, 2015, Sices *et al*. 2007).

Findings of higher rates of literacy difficulties in cases where SSD co‐occurs with language problems have led to the suggestion that linguistic abilities may interact with phonological deficits in literacy acquisition (Apel and Lawrence 2011, Peterson *et al*. 2009, Sices *et al*.; 2007). For example, Apel and Lawrence (2011) found that in a sample of forty‐four 6–8‐year‐old children with SSD and no history of language impairment, children with SSD performed below typically developing controls on both phonological awareness and morphological awareness measures. Morphological, rather than phonological, awareness was a significant predictor of spelling. They concluded that children with SSD may have an underlying problem with general linguistic awareness which places them at risk for problems with literacy development, in the absence of any identified history of language impairment.

Age also appears to be important. Those who have included older age groups in their investigations have suggested that SSD status becomes less important as the child ages. Lewis *et al*. (2000) found that 18% of children with SSD had difficulties with reading at age 8–12 years compared with 75% who had SSD and comorbid language difficulties, while Hayiou‐Thomas *et al*. (2017) found that SSD status accounted for up to 5.8% of the variance in phoneme awareness and spelling at age 6 years, but only 1.9% of the variance in reading and spelling at age 8 years, suggestive of a short‐term impact on literacy. There is a need to see whether these patterns are also observed in larger samples of older children.

In their systematic review, McCormack *et al*. (2009) identified that variation in terminology is a key limitation in the current evidence‐base for literacy outcomes of SSD, making it hard to make comparisons across studies. Whilst some studies have included children from community samples or used longitudinal designs (e.g., Hayiou‐Thomas *et al*. 2017, Peterson *et al*. 2009), most have focused on SSD in younger childhood rather than PSD using small, clinic‐referred samples (Leitao and Fletcher 2004, Lewis *et al*. 2000, Preston and Edwards 2007, Rvachev 2007), many restricted to pre‐school children in cross‐sectional studies (e.g., Anthony *et al*. 2011, Holm *et al*. 2008, Preston and Edwards 2010). Thus, there is a need for prospective, longitudinal studies of large community samples of older children with and without PSD to address long‐term outcomes (Johnson *et al*. 2010, Law *et al*. 2000).

### PSD and educational attainment

Educational attainment is associated with progress in the development of literacy skills (Gottfried *et al*. 2015, McCoach *et al*. 2017), which in turn is associated with SSD. It is therefore reasonable to expect that children who exhibit PSD are likely to do worse at school. This link with educational attainment is well established for children with developmental language disorder (e.g., Conti‐Ramsden *et al*. 2009, 2018, Snowling *et al*. 2001) and is pivotal to the campaign to ensure better resources and support for children with developmental language disorder in school. There is similar evidence from large‐scale population‐based studies for children with speech and language needs identified in early childhood by parent or teacher report (Harrison *et al*. 2009, McLeod *et al*. 2019). Fewer studies have addressed educational attainment for speech difficulties and those that have, focus on children with SSD rather than older children with PSD. In the United States, Shriberg and Kwiatkowski (1988) found that more than 80% of children with SSD identified in their preschool years required additional support whilst at school. McCormack *et al*. (2009), in a systematic review of activity and participation outcomes for children with SSD, found some evidence for poor outcomes in attention and thinking (such as information processing and reasoning skills) and mathematical skills, in addition to reading and writing. Moreover, adults with a history of SSD, characterized as phonological disorder, have been found to achieve lower grades and complete fewer years of formal education in comparison with typically developing adults (Felsenfeld *et al*. 1994).

Further evidence for the impact on educational attainment can be obtained from Statutory Assessment Test (SAT) results. In England, these are carried out routinely in schools at the age of 7 (Key Stage 1) and 11 years (Key Stage 2). National data indicate that many children identified as having ‘speech language and communication needs’ (SLCN) perform below expected levels. For example, in Lindsay *et al*. (2010), only 25% of pupils with SLCN at Key Stage 2 obtained the expected level in English, 29% in mathematics and 45% in science compared with 79%, 76% and 87% nationally, respectively. SLCN is one of the categories of SEN used in the English education system. Being categorized as SEN enables access to additional support within school and from external agencies. However, the category of SLCN encompasses children with a wide range of different profiles. Moreover, data have shown that around one in five children initially identified as having SLCN are likely to transition to another SEN category as they progress through school and are most likely to change to ‘specific learning difficulty’ or ‘moderate learning difficulty’ (Meschi *et al*. 2012). It is possible, therefore, that older children with PSD are ‘hidden’ in SEN figures if associated difficulties such as cognition and learning needs are recorded as their primary impairment.

Nathan *et al*. (2004b) investigated the link between pre‐school SSD and SATs performance at age 7 years in a clinical sample of 39 children and found that, overall, children with a history of SSD performed more poorly than controls on reading, spelling and mathematics. When children with PSD and resolved SSD were analysed separately, the PSD group scored markedly below controls on mathematics, reading, reading comprehension and spelling. There was no evidence that the resolved SSD group performed differently to controls.

### Study aims

The current evidence base suggests that children with PSD are more likely to struggle with the acquisition of literacy skills and are less likely to do well on measures of educational attainment. However, the published studies have used clinical samples or identified the population of interest through teacher or parent report, sometimes using a broader categorization of speech, language and communication needs or SSD rather than PSD specifically. Large‐scale prospective community‐based studies, which include all children—not just those who have been referred to clinical services—and involve direct assessment of children's speech, are needed to examine the long‐term educational outcomes for children with PSD and to determine if they are more likely than their peers to receive additional support at school through being identified as having SEN.

The Avon Longitudinal Study of Parents and Children (ALSPAC) is a large prospective population‐based study that has collected data on children's speech development and long‐term education outcomes across primary and secondary schools. This study aimed to identify whether children identified with PSD at age 8 years performed worse in comparison with their peers on educational outcomes. Two specific questions were addressed:

- How do children identified with PSD at age 8 years perform on educational attainment tests at ages 10–11 and 13–14 years in comparison with children without PSD?
- Are children identified with PSD at age 8 years more likely to receive a label of SEN and, if so, which SEN category is most commonly used?

The answers to these questions will help determine whether children with PSD are at greater risk of low educational outcomes than their peers, and whether and how their needs are being identified through categorization of SEN.

## Method

ALSPAC (www.bristol.ac.uk/alspac) is a transgenerational observational population‐based study of health and development across the life span, which enrolled mothers in early pregnancy in Bristol and surrounding areas in England in the period 1991–92. The initial recruitment resulted in a core cohort of 14,541 pregnancies and 13,988 children alive at 12 months. ALSPAC has detailed information on parents (Fraser *et al*. 2013) and children (Boyd *et al*. 2013), collected prospectively at multiple times during pregnancy and throughout childhood. Sources of data include self‐report surveys, clinical assessments, birth, medical and educational records, and biological samples. Since the children were aged 7 years, the entire cohort was invited to attend for direct assessment of varying aspects of development at regular intervals (known as the ‘Focus’ clinics). The second of these Focus clinics was the ‘Focus at 8’ clinic in which speech was assessed.

The study website contains details of the data that are available through a fully searchable data dictionary and variable search tool (http://www.bristol.ac.uk/alspac/researchers/our-data/).

Ethical approval for the study was obtained from the ALSPAC Ethics and Law Committee and the Local Research Ethics Committees (http://www.bristol.ac.uk/alspac/researchers/research-ethics/). Informed consent for the use of data collected via questionnaires and clinics was obtained from participants following the recommendations of the ALSPAC Ethics and Law Committee at the time.

### Participants

The sample for this study was the 7390 ALSPAC child participants who attended the research clinic at age 8 years. In the clinic, a sample of continuous speech was collected by trained assessors, who noted whether the children's speech was atypical, beyond what could be accounted for by accent variation. A total of 991 (13.4%) children were identified as having some unusual features in their speech which could qualify them as atypical; 580 (7.8%) of these were identified as having common clinical distortions as defined by Shriberg (1993). As these speech errors are not associated with poor outcomes in education (Shriberg 2010), this group was excluded from further analysis. The continuous speech samples of the remainder of those identified as having atypical speech (*N* = 411, 5.5%) were transcribed and analysed by qualified speech and language therapists using the PROPH programme from the software Computerized Profiling (Long *et al*. 2006). The recordings from a random sample of 50 children from the rest of the cohort were also transcribed and analysed using this approach to provide normative data.

**Figure 1:**
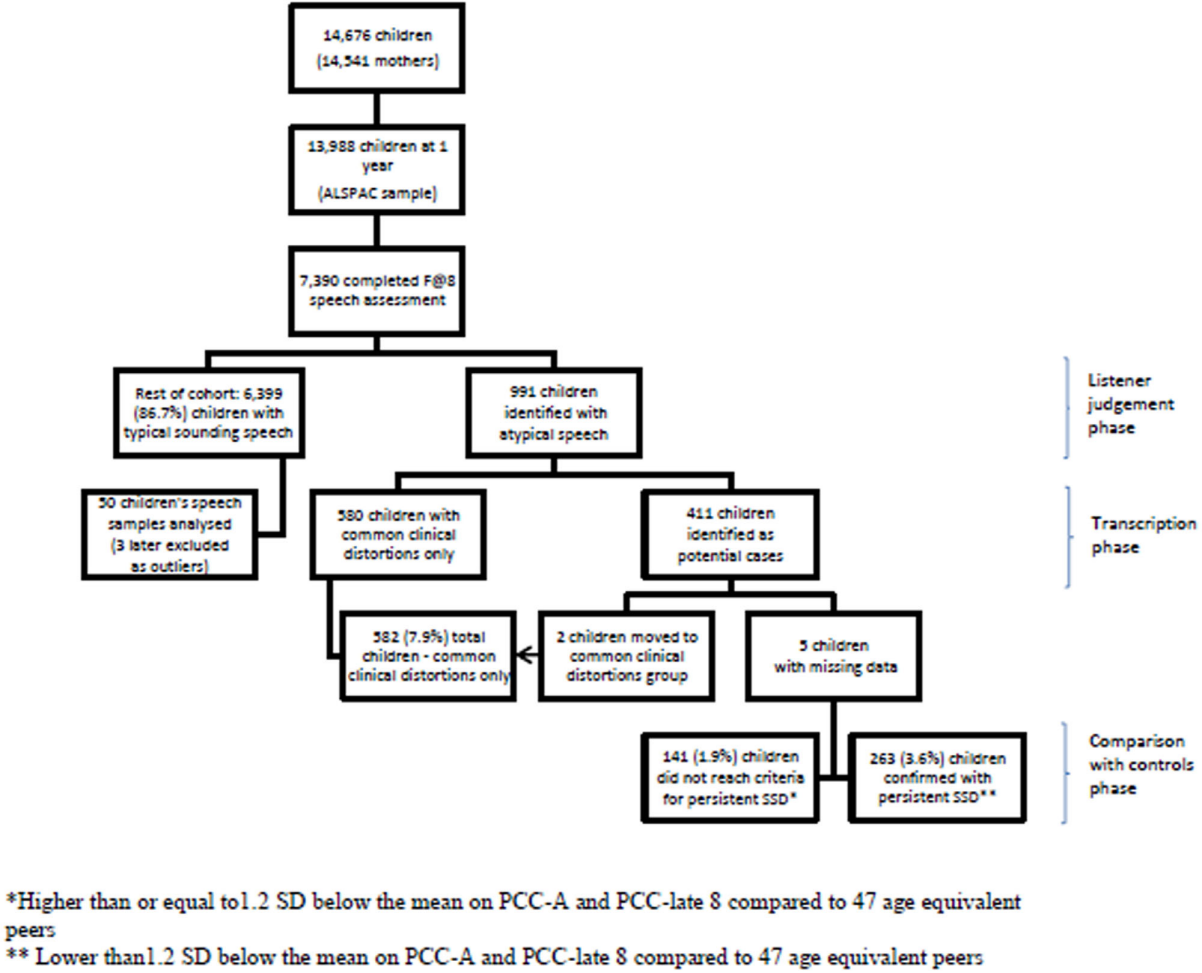
Summary for case identification. [Color figure can be viewed at wileyonlinelibrary.com]

The means and standard deviations (SD) for the control sample were calculated for two measures of percentage consonants correct (PCC): PCC‐late 8 (the last eight consonants typically acquired in the development sequence as identified by Shriberg *et al*. 1997) and PCC‐Adjusted, which counts all common clinical distortions as acceptable (Shriberg *et al*. 1997). Those children in the atypical speech group whose scores for PCC‐late 8 and PCC‐Adjusted were lower than −1.2 SD below the mean of the control group were confirmed as having PSD and became the case group (*N* = 263, 3.6%). Of the remainder (*N* = 148), five had missing data and two demonstrated only common clinical distortions on transcription and so joined the excluded group. The remaining children (*N* = 141, 1.9%) were also excluded from further analysis, as comparison of this group alongside the case and control groups showed that there were distinct differences in the profiles of children in the groups with regards to demographic, cognitive and speech motor skills (Wren *et al*. 2012). The total sample size for this study was therefore 6662 children, consisting of a case group of children with confirmed PSD (*N* = 263, 3.6%) and a control group made of the rest of the cohort but excluding those with speech errors confined to common clinical distortions and those whose speech errors were not sufficient to reach criteria for case status (*N* = 6399) (figure 1). Further information on the process for identifying the case group is available in Wren *et al*. (2016).

### Outcome measures

Two types of outcome measures were used in this study: educational attainment and SEN categorization. Educational attainment was measured using data from school standard attainment tests carried out in England at the end of primary education (end of Key Stage 2, ages 10–11 years) and before starting courses for national examinations in secondary education (end of Key Stage 3, ages 13–14 years). Performance of cases was compared with that of controls. For each subject (English, mathematics and science), the target level for children at Key Stage 2 is 4 and the target level at Key Stage 3 is 5. Children scoring less than these targets at each age are considered to be underachieving. A variable was also created for underachieving in any of the three individual subjects to provide more power with the numbers available.

Data on the sample's SEN categorization were obtained between ages 11 and 13 years from the Pupil Level Annual Schools Census (PLASC) across three categories of SEN: cognition and learning needs; behaviour, emotional and social difficulties; and communication and interaction needs. Children were considered to have SEN if they had a record of: ‘school action plus’, where help external to the school had been requested to support the child; ‘school action plus and statutory assessment’, where children were being assessed for a statement of SEN; or ‘statement of special educational needs’, where a statement which outlined the child's needs and corresponding provision had been awarded.

Availability of outcome data varied. Some data were only available for a subsample (Key Stage 2 data). Other data were limited to what could be obtained through linkage of the ALSPAC data set with educational PLASC data.

### Potential confounders

Several measures associated with educational attainment were included as potential confounders in this study. Biological sex, as recorded in midwifery birth records, and socioeconomic status, as measured by highest level of maternal education reported by the mother in the questionnaire completed at 32 weeks’ gestation, were used in all adjusted analyses. Data on biological sex and maternal education were limited to those participants whose parents had provided this information.

In addition, verbal and performance subtests as well as total IQ scores for the Wechsler Intelligence Scale for Children (WISC; Wechsler *et al*. 1992) were included in sub‐analyses. The verbal subtests included five subtests covering information (assessing the child's knowledge), similarities (explaining similarities between things, e.g., red and blue), mental arithmetic, receptive vocabulary and comprehension. The combined score from these subtests were used as a proxy for oral language skill. The performance subtests included another five subtests comprising picture completion tasks (where the child points out what is missing from pictures), coding (where shapes corresponding to different numbers are copied), picture arrangements (where pictures must be ordered to make a meaningful sequence), block design (pictures of patterns of blocks are copied with real blocks) and object assembly (putting puzzles together). The combined score for these subtests were used as a proxy for non‐verbal IQ. These measures were collected on the same day as the speech research clinic session. Not all children attended both the speech and the IQ session and therefore only data on children who attended both were used in the analysis.

### Statistical analysis

Logistic regression analyses were conducted to compare outcomes for the case group (children classified as having PSD, *N* = 263) with controls (the rest of the cohort, as defined above, *N* = 6399). Unadjusted and adjusted odds ratios with 95% confidence intervals (CIs) are presented.

Adjustment was made for biological sex and maternal education (model 1), for performance IQ (model 2a) and verbal IQ (model 2b) and finally for total IQ (model 3).

At each point, all available data were used. All analyses were conducted using Stata (Version 13; Stata Corp, TX, USA).

## Results

Table 1 summarizes the outcome measures of interest, indicating the source and the age they were collected and the available sample size. Compared with the baseline cohort, those children who were assessed at the 8‐year clinic were more likely to be female and to have mothers with higher levels of education.

**Table 1:** Summary of variables and sample size

Variable	Variable type	Variable source	Age (years) when collected	Did not achieve the target level/total sample—controls	Did not achieve the target level/total sample—cases
*Academic achievement*					
Key Stage 2 English	Outcome	Standard Attainment Test	10–11	88/972 (9.05%)	12/51 (23.53%)
Key Stage 2 Maths	Outcome	Standard Attainment Test	10–11	99/972 (10.18%)	11/51 (21.57%)
Key Stage 2 Science	Outcome	Standard Attainment Test	10–11	46/972 (4.73%)	5/51 (9.8%)
Key Stage 2 Combined	Outcome	Standard Attainment Test	10–11	138/972 (14.2%)	17/51 (33.33%)
Key Stage 3 English	Outcome	Standard Attainment Test	13–14	797/5115 (15.58%)	64/211 (30.33%)
Key Stage 3 Maths	Outcome	Standard Attainment Test	13–14	713/5115 (13.94%)	51/211 (24.17%)
Key Stage 3 Science	Outcome	Standard Attainment Test	13–14	772/5115 (15.09%)	48/211 (22.75%)
Key Stage 3 Combined	Outcome	Standard Attainment Test	13–14	1204/5115 (23.54%)	79/211(37.44%)

*Special educational needs categorization*				*Label given/total sample—controls*	*Label given/total sample—cases*
Cognition and learning needs	Outcome	SEN categorization	11–13	122/5401 (2.26%)	24/225 (10.67%)
Behavioural, emotional and social difficulties	Outcome	SEN categorization	11–13	44/5401 (0.81%)	4/225 (1.78%)
Communication and interaction needs	Outcome	SEN categorization	11–13	25/5401 (0.46%)	10/225 (4.44%)
Any SEN (excluding sensory)	Outcome	SEN categorization	11–13	185/5401 (3.43%)	36/225 (16%)

*Confounders*				*Number available—controls*	*Number available—cases*
Gender	Confounder	Midwifery records	Antenatal	6399	263
Highest level of maternal education reported by the mother	Confounder	Mother's questionnaire at 32 weeks gestation	Antenatal	5924	242
Performance IQ (subtest of WISC)	Confounder	Wechsler Intelligence Scale for Children	8‐year clinic	6309	258
Verbal IQ (subtest of WISC)	Confounder	Wechsler Intelligence Scale for Children	8‐year clinic	6319	257
Combined IQ (total of WISC)	Confounder	Wechsler Intelligence Scale for Children	8‐year clinic	6292	256

Note: SEN, special educational needs; IQ, intelligence quotient; WISC, Wechsler Intelligence Scale for Children.

### Educational attainment

Table 2 presents the results for case children compared with the control group in terms of not achieving the age‐appropriate target level on the standard attainment tests at the end of Key Stages 2 and 3. The odds ratio gives the increase in odds of *underachieving* for a case child compared with a control child.

**Table 2:** Academic achievement outcomes: Odds ratios (95% confidence intervals (CI)) for not achieving target levels in standard educational tests ages at Key Stages (KS) 2 and 3^a^ for cases versus controls

	Model 0: Unadjusted		Model 1: Adjusted for biological sex and maternal education		Model 2a: Adjusted for biological sex, maternal education and performance IQ		Model 2b: Adjusted for biological sex, maternal education and verbal IQ		Model 3: Adjusted for biological sex, maternal education, verbal and performance IQ	
Outcome	OR [95% CI]	*p‐*value	OR [95% CI]	*p‐*value	OR [95% CI]	*p‐*value	OR [95% CI]	*p‐*value	OR [95% CI]	*p‐*value
KS2 level 4+ in English	3.09 [1.56, 6.12]	0.001	3.30 [1.58, 6.89]	0.001	2.24 [0.98, 5.13]	0.055	2.88 [1.23, 6.75]	0.015	2.36 [0.98, 5.67]	0.056
KS2 level 4+ in Maths	2.43 [1.21, 4.88]	0.013	2.59 [1.20, 5.59]	0.016	1.70 [0.71, 4.05]	0.232	2.12 [0.86, 5.21]	0.102	1.73 [0.68, 4.35]	0.249
KS2 level 4+ in Science	2.19 [0.83, 5.77]	0.113	2.25 [0.81, 6.23]	0.121	1.20 [0.36, 3.99]	0.768	1.46 [0.44, 4.85]	0.54	1.14 [0.33, 3.93]	0.84
KS2 level 4+ in all three subjects	3.02 [1.64, 5.56]	< 0.0001	3.34 [1.70, 6.55]	< 0.0001	2.44 [1.12, 5.32]	0.025	3.17 [1.44, 7.01]	0.004	2.60 [1.13, 5.99]	0.025
KS3 level 5+ in English	2.36 [1.74, 3.19]	< 0.0001	1.99 [1.42, 2.79]	< 0.0001	1.75 [1.22, 2.51]	0.002	1.50 [1.03, 2.19]	0.034	1.48 [1.01, 2.17]	0.042
KS3 level 5+ in Maths	1.97 [1.42, 2.72]	< 0.0001	1.78 [1.25, 2.54]	0.002	1.48 [1.00, 2.18]	0.053	1.21 [0.81, 1.82]	0.361	1.18 [0.78, 1.79]	0.429
KS3 level 5+ in Science	1.66 [1.19, 2.31]	0.003	1.48 [1.03, 2.13]	0.036	1.18 [0.78, 1.77]	0.434	0.95 [0.62, 1.44]	0.795	0.92 [0.60, 1.41]	0.687
KS3 level 5+ in all three subjects	1.94 [1.46, 2.59]	< 0.0001	1.67 [1.21, 2.29]	< 0.0001	1.45 [1.02, 2.05]	0.037	1.21 [0.84, 1.73]	0.317	1.18 [0.81, 1.71]	0.388

Note: ^a^KS2 ages 10–11 years; KS3 ages 13–14 years.

*N* for KS2: model 0 = 1023; model 1 = 932; model 2a = 910; model 2b = 908; and model 3 = 905.

*N* for KS3: model 0 = 5326; model 1 = 4917; model 2 = 4849; model 2b = 4852; and model 3 = 4833.

The unadjusted models (model 0) show that for all subjects, underachievement was more likely in cases compared with controls, but this was stronger for English at Key Stages 2 and 3 (unadjusted OR = 3.09 [95% CI = 1.56–6.12] and 2.36 [95% CI = 1.74–3.19]), respectively.

After adjustment for biological sex and maternal education (model 1), the odds ratios increased for underachieving for Key Stage 2 English (OR = 3.30 [95% CI = 1.58–6.89]), and for all three subjects at this level (OR 3.34 [95% CI = 1.70–6.55]). The odds ratios were attenuated for Key Stage 3, but strong associations remained.

After further adjustment for performance IQ (model 2a), the strengths of all these associations were attenuated at both time points.

In comparison, further adjustment for verbal IQ only (model 2b) resulted in a different pattern of attenuation. Stronger associations were observed for Key Stage 2 compared with Key Stage 3, with English in particular showing a moderate association (this was weaker when only performance IQ was included) (OR = 2.88 [95% CI = 1.23–6.75]) and OR = 2.24 [95% CI = 0.98–5.13] after adjustment for verbal and performance IQ, respectively). Not achieving the target level in all three subjects was much more likely at Key Stage 2. No associations were shown for mathematics and science at Key Stages 2 or 3 and no association was shown for all three subjects at Key Stage 3.

The last model (model 3) adjusted for both verbal and performance IQ using the total WISC score together with biological sex and maternal education. In this model, only two associations remained: English at Key Stage 3 (OR = 1.48 [95% CI = 1.01–2.17]) and all three subjects at Key Stage 2 (OR = 2.60 [95% CI = 1.13–5.99]). The latter was itself primarily driven by not achieving the target in English, which was further attenuated after full adjustment (OR = 2.36 [(95% CI = 0.98, 5.67]). This suggests that IQ was important in explaining the variance in educational attainment as measured by standardized attainment tests, with verbal IQ having more impact in the older age range whereas performance IQ had an impact in the younger age band.

### Special educational needs (SEN)

Table 3 presents the associations between having a label of SEN in cases versus controls. As with the educational attainment data, five models are reported, one showing unadjusted data and four showing different adjustments.

**Table 3:** Special educational needs (SEN) status outcomes: Odds ratios (95% confidence intervals (CI)) for having SEN status in cases versus controls

	Model 0: Unadjusted		Model 1: Adjusted for biological sex and maternal education		Model 2a: Adjusted for biological sex, maternal education and performance IQ		Model 2b: Adjusted for biological sex, maternal education and verbal IQ		Model 3: Adjusted for biological sex, maternal education, verbal and performance IQ	
Outcome	OR [95% CI]	*p‐*value	OR [95% CI]	*p‐*value	OR [95% CI]	*p‐*value	OR [95% CI]	*p‐*value	OR [95% CI]	*p‐*value
Cognition and learning needs	5.17 [3.26, 8.18]	< 0.0001	4.33 [2.60, 7.20]	< 0.0001	3.61 [2.10, 6.22]	< 0.0001	3.12 [1.80, 5.41]	< 0.0001	3.05 [1.74, 5.33]	< 0.0001
Behavioural, emotional and social difficulties	2.20 [0.80, 6.19]	0.134	1.36 [0.41, 4.45]	0.616	1.38 [0.42, 4.60]	0.595	1.29 [0.39, 4.30]	0.676	1.30 [0.39, 4.33]	0.671
Communication and interaction needs	10.00 [4.74, 21.09]	< 0.0001	6.96 [3.05, 15.87]	< 0.0001	4.69 [1.83, 12.01]	0.001	4.26 [1.63, 11.15]	0.003	4.06 [1.56, 10.82]	0.004

Note: *N* for SEN: model 0 = 5626; model 1 = 5197; model 2a = 5123; model 2b = 5128; and model 3 = 5106.

A strong association was observed for ‘cognition and learning needs’ for all models except that which included total IQ score. Adjusting for verbal IQ attenuated the odds ratio to a greater extent than adjusting for performance IQ (OR = 3.12 [95% CI = 1.80, 5.41] compared with OR = 3.61 [95% CI = 2.10, 6.22]). In contrast, the strongest evidence for an association shown for a label of ‘behavioural, emotional and social difficulties’ was for model 3 (i.e., adjusting for both performance and verbal IQ).

Finally, there were consistently strong associations seen between PSD status and having a label of ‘communication and interaction needs’. Even after full adjustment cases were four times more likely to be given this label compared with controls.

## Discussion

This study used data from a large community population study to investigate educational outcomes for children with PSD. Using data from English school standard attainment tests carried out at the end of primary education (end of Key Stage 2) and before starting courses for national examinations in secondary education (end of Key Stage 3), we have shown that children with PSD at age 8 years were more likely to achieve lower attainment scores at both time points in English and mathematics, and across all three subjects of English, mathematics and science at Key Stage 3, even after controlling for biological sex and maternal education. Specifically, children with PSD at age 8 years were more than three times more likely to score below target levels in English and 2.6 times more likely to score below target levels in mathematics at ages 10–11 years. This was maintained at ages 13–14 years, at which point children with PSD at age 8 years were still nearly twice as likely to score below target levels in English and mathematics. While there was no evidence of an association between PSD and underachievement in science at ages 10–11 years, by ages 13–14 years children with PSD at age 8 years were 1.5 times more likely to score below target levels. These results have important implications for educational attainment in older childhood, for example, UK standard attainment test scores at Key Stage 2 have been found to correlate highly with scores in later secondary school examinations at age 16 years (Strand 2006).

After controlling for verbal IQ, children with PSD were still more likely to score below target levels for English at both time points. Controlling for performance IQ showed the same pattern at Key Stage 3. This was not the case for mathematics or science (associations were not significant after correction for verbal, performance or full‐scale IQ at either time‐point). This might also help to explain the difference in findings for science at ages 10–11 versus 13–14 years. While the unadjusted odds ratio shows evidence of an association in the older age group, this is not apparent following adjustment for verbal and performance and total IQ, suggesting that at this age, IQ rather than SSD status is important. When attainment for English, mathematics and science were combined, children with PSD were more likely to score below target levels at Key Stage 2 after controlling for verbal, performance and full‐scale IQ, and at Key Stage 3 after controlling for performance IQ.

These results are consistent with previous studies which have shown a relationship between SSD and literacy levels or standard attainment test performance in younger children (Anthony *et al*. 2011, Felsenfeld *et al*. 1994, Holm *et al*. 2008, Lewis *et al*. 2000, 2007, McCormack *et al*. 2009, Nathan *et al*. 2004a, Overby *et al*. 2012, Preston and Edwards 2007). However, the research reported in this paper shows that these problems persist into older childhood (ages 10–11 years) and early adolescence (ages 13–14 years) and are visible in a population sample which includes children who have not accessed clinical services and therefore would not be included in previous research which has used data from clinical populations only (Lewis *et al*. 2000, Preston and Edwards 2007). Moreover, this research has shown that the relationship exists after adjustment for verbal IQ. This contrasts with previous findings (Hayiou‐Thomas *et al*. 2017, Lewis *et al*. 2000) and suggests that PSD can have an important impact on children's literacy development beyond the early years of reading and writing instruction.

The exclusion of children with isolated speech errors, sometimes referred to as common clinical distortions and consistent with Shriberg's (1993) definition of children whose problems are limited to distortion errors on sibilants and/or liquids (Shriberg 2010), differs from other studies in the field such as Tambyraja *et al*. (2020) which employed a broader definition of SSD. Shriberg (2010) argued that these errors are not associated with adverse outcomes in education. Moreover, Wren *et al*. (2012) showed that children whose speech errors were restricted to common clinical distortions were similar to controls on tasks of non‐word repetition and significantly different from children with PSD on this same measure. Poor non‐word repetition has long been associated with difficulties with literacy skills (Snowling 1981), suggesting that had children whose problems were limited to common clinical distortions been included in this analysis, associations with academic performance may have been weaker, or indeed non‐existent. In Tambyraja *et al*. (2020), 25% of their sample of children with SSD, including children who may have had speech sound distortions limited to common clinical distortions, showed concomitant problems with word decoding. However, an association specifically with PCC scores was not demonstrated. An interesting additional analysis of these data would be to explore with the prevalence of 25% is increased if children with common clinical distortion errors only were excluded, and indeed if this also resulted in an association between PCC score and word decoding.

Our second objective was to investigate associations between PSD at age 8 years and categories of SEN at ages 11–13 years. Strong associations were found showing that children with PSD at age 8 years were more likely to receive a label of SEN in secondary school (seven times more likely to be identified with communication and interaction needs and over four times more likely to be identified with cognition and learning needs after adjusting for biological sex and maternal education between ages 11 and 13 years). After further adjustment for verbal or performance IQ, children with PSD were still more likely to be categorized as having communication and interaction needs and cognition and learning needs. This supports previous findings that SSD is linked to difficulties with learning and cognition (Felsenfeld *et al*. 1994, Nathan *et al*. 2004b).

The association between PSD and the SEN category of cognition and learning is consistent with findings that the prevalence of children identified with speech, language and communication needs as their primary need decreases from ages 5 to 16 years (Lindsay and Strand 2016) and that children with identified speech, language and communication needs at an earlier age will transition to different SEN categories later on and are more likely to be identified as having moderate or specific learning difficulties than behavioural, emotional or social difficulties in secondary school (Meschi *et al*. 2012). Another explanation is that difficulties with cognition and learning are linked to a fundamental difficulty with speech processing that limits some of their progress in school (Stackhouse and Wells 1997).

### Limitations and future research

This study was limited by the comparatively low sample size of children with PSD and available data on educational attainment at Key Stage 2. However, given that outcome data for Key Stage 4 and SEN were obtained from linked education records the amount of missing data in those with PSD is minimal.

Findings from studies that have compared SSD with SSD and language difficulties have suggested that there may be increased risk of difficulties with phonological awareness and later literacy when SSD co‐occurs with language problems (Bishop and Clarkson 2003, Hayiou‐Thomas *et al*. 2017, Lewis *et al*. 2007, 2015, McCormack *et al*. 2009, Nathan *et al*. 2004a, Peterson *et al*. 2009). While we did not exclude children with co‐morbid language difficulties from our sample, we did adjust for verbal IQ scores which are based on tests involving language skills.

Associations with outcome and the type of speech error was beyond the scope of this study, however this would be an interesting area for future research given the links that have been found between atypical errors and both persistence of SSD (Morgan *et al*. 2017, Dodd *et al*. 2017) and literacy development (Dodd *et al*. 1995, Holm *et al*. 2008, Leitao *et al*. 2000, Leitao and Fletcher 2004, Preston and Edwards 2010).

## Conclusions

This study provides further support for the association between SSD and later educational outcomes. Specifically, this large‐scale population‐based data set has shown that PSD at age 8 years is associated with poorer attainment at ages 10–11 and 13–14 years in the core subject of English. Moreover, children with PSD are more likely to be identified with SEN at ages 11–13 years, including cognition and learning needs, and communication and interaction needs. These findings have important implications for later life with the potential for impact on educational attainment at school leaving age and consequences for future life chances. It is important that we recognize the risks for this population and provide ongoing support throughout primary education and into the secondary school years and ensure that the focus of services on early years provision does not limit what is available for older age groups.
